# Hemagglutinin-esterase-fusion (HEF) protein of influenza C virus

**DOI:** 10.1007/s13238-015-0193-x

**Published:** 2015-07-28

**Authors:** Mingyang Wang, Michael Veit

**Affiliations:** Institute of Virology, Veterinary Medicine, Free University Berlin, Berlin, Germany

**Keywords:** influenza C virus, HEF, structure, receptor binding, esterase, membrane fusion

## Abstract

Influenza C virus, a member of the *Orthomyxoviridae* family, causes flu-like disease but typically only with mild symptoms. Humans are the main reservoir of the virus, but it also infects pigs and dogs. Very recently, influenza C-like viruses were isolated from pigs and cattle that differ from classical influenza C virus and might constitute a new influenza virus genus. Influenza C virus is unique since it contains only one spike protein, the hemagglutinin-esterase-fusion glycoprotein HEF that possesses receptor binding, receptor destroying and membrane fusion activities, thus combining the functions of Hemagglutinin (HA) and Neuraminidase (NA) of influenza A and B viruses. Here we briefly review the epidemiology and pathology of the virus and the morphology of virus particles and their genome. The main focus is on the structure of the HEF protein as well as on its co- and post-translational modification, such as N-glycosylation, disulfide bond formation, S-acylation and proteolytic cleavage into HEF1 and HEF2 subunits. Finally, we describe the functions of HEF: receptor binding, esterase activity and membrane fusion.

**Electronic supplementary material:**

The online version of this article (doi:10.1007/s13238-015-0193-x) contains supplementary material, which is available to authorized users.

## INTRODUCTION

Influenza C virus was first isolated during an epidemic of respiratory illness in 1947. Since the virus showed no cross reactivity with antisera against influenza A and B viruses it was classified as a new genus of the *Orthomyxoviridae*, named influenza C virus (Francis et al., 1950; Taylor, 1949, 1951). Influenza C virus usually causes inflammation of the upper respiratory tract, especially in children from two to six years of age. Clinical symptoms, such as cough, fever, malaise are typically mild. Only occasionally the virus spreads to the lower respiratory tract and causes bronchitis, bronchiectasie and broncho-pneumonia (Gouarin et al., 2008; Kauppila et al., 2014; Matsuzaki et al., 2007; Matsuzaki et al., 2006; Muraki and Hongo, 2010). Although influenza C virus infections occur primarily in a pattern of sporadic cases or in limited outbreaks (Joosting et al., 1968; Minuse et al., 1954), serological studies indicated that this virus is widely distributed around the world and that the majority of humans develope antibodies against the virus early in life (Matsuzaki et al., 2006; Salez et al., 2014). In a serological study carried out in France, 60%–70% of the population was found to be previously exposed to the virus, the highest rates of positive samples was found in the 16–30 years age group (Manuguerra et al., 1992). In a 6-year tracking study in hospitalized children in Spain, influenza C infections accounted for 13% of influenza-positive cases (Calvo et al., 2013). The results indicate intense circulation of influenza C virus in the human population.

The primary host and reservoir of influenza C virus are humans, but there is evidence that this virus possesses the ability to also infect animals (Muraki and Hongo, 2010). Serological studies showed that antibodies against influenza C virus are widely present in dogs and especially in pigs (Brown et al., 1995; Horimoto et al., 2014; Manuguerra and Hannoun, 1992; Manuguerra et al., 1993; Ohwada et al., 1987; Yamaoka et al., 1991; Youzbashi et al., 1996). In 1981, a number of influenza C virus strains were isolated from pigs in Beijing and these strains could be transmitted from pig to pig under experimental conditions (Guo et al., 1983).

In 2011, an influenza C-like virus was isolated from clinically ill pigs exhibiting influenza-like symptoms (C/Oklahoma/1334/2011) and also from cattle (D/bovine/Oklahoma/660/2013) which subsequently turned out to be the main reservoir of this newly discovered virus (Collin et al., 2014, 2015; Hause et al., 2013). Phylogenetic analysis showed that these strains have only 50% overall amino acid homology to human influenza C viruses, a divergence similar to that described between influenza A and B viruses (Hause et al., 2013). Accordingly, no cross reactivity was observed between these strains and human influenza C virus antisera. This new strain has a broader cell tropism than human influenza C virus and is capable of infecting and transmitting by direct contact in both pigs and ferrets. It also encodes a novel mechanism for generating the M1 protein and, importantly, is unable to reassort with human influenza C virus and generate viable progeny. Based on these differences to influenza C virus it was suggested that this virus warrants classification as a new genus of influenza virus, named influenza D virus (Collin et al., 2014; Hause et al., 2013).

## VIRAL PARTICLE AND GENOME STRUCTURE OF INFLUENZA C VIRUS

Influenza C virus particles exhibit two morphologies, either spherical with a diameter of 80–120 nm or filamentous with the same diameter but with lengths in µm range (Waterson et al., 1963). Already during the budding process at the plasma membrane, filamentous particles may aggregate via their long axes into 500 μm long cord-like structures, which are all covered by a layer of surface projections (Nishimura et al., 1990). Studies using reverse genetics showed that an amino acid exchange at residue 24 of the M1 protein (Ala to Thr) that reduces membrane association of this intrinsically hydrophobic protein eliminates cord formation and also affects virus morphology (Muraki et al., 2004; Nishimura et al., 1994). Another unique characteristic of influenza C virus particles observed by electron microscopy is a reticular hexagonal structure, which is formed by the HEF protein and discussed in more detail below (Apostolov and Flewett, 1969; Flewett and Apostolov, 1967; Herrler et al., 1981).

**Figure 1:**
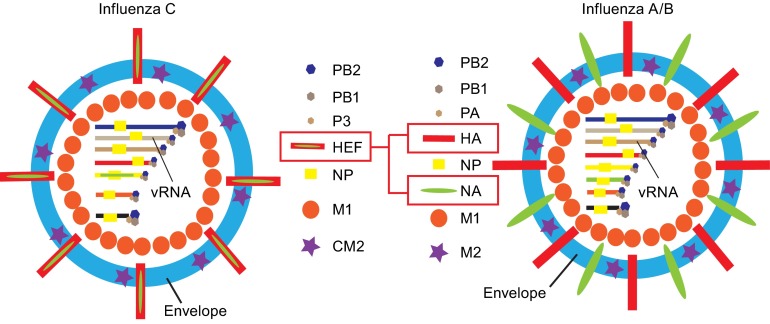
**Scheme of influenza C virus and influenza A/B virus particles**. Proteins having the same function are depicted with the same symbol. Note that influenza C virus has only one spike protein, the hemagglutinin-esterase-fusion glycoprotein HEF that combines the functions of both hemagglutinin (HA) and neuraminidase (NA) from influenza A and B virus. PB1, PB2, P3 and PB1, PB2, PA are the polymerase proteins of influenza C virus and influenza A/B virus, respectively, that build together with the nucleoprotein NP and the viral RNA-segments the ribonucleoprotein complexes (vRNP). M1 is the matrix protein and M2 and CM2 the proton-channel

**Figure 2:**
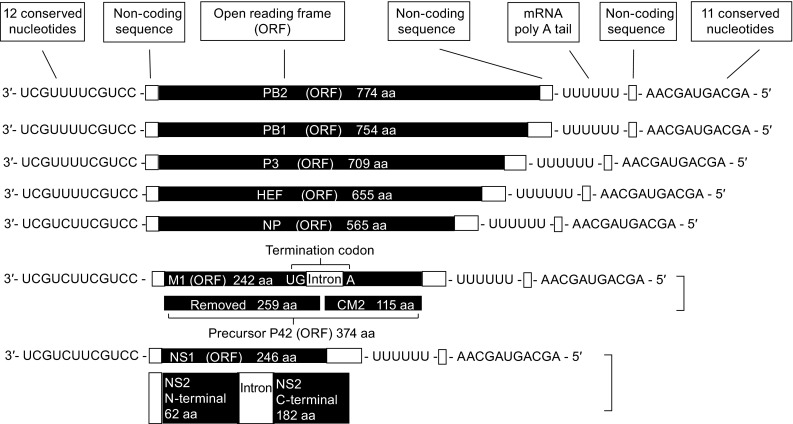
**Structure of the seven influenza C virus genome segments**. Open reading frames (ORFs) are indicated in black. Segment six and seven encode two proteins which are generated by splicing. Influenza C virus possesses 7 minus-senses, single-strand and segmented RNA. Each segment possesses 12 conserved nucleotides at 3′ terminal and 11 conserved nucleotides at 5′ terminal. A poly U motif is close to 5′ terminal and it transcripts into mRNA poly A tail. Each of the longest 5 segments possesses only 1 open-reading-frame (ORF) and encodes PB2, PB1, P3, HEF and NP, respectively

The influenza C virus genome consists of negative-sense, single-stranded RNA (Desselberger et al., 1980), but in contrast to influenza A and B virus only seven (not eight) gene segments are present in virus particles (see Fig. 1 for the structure of a virus particle and Fig. 2 for the structure of viral genome segments). The longest three segments encode the proteins PB2, PB1 and P3 that form the heterotrimeric polymerase complex (Yamashita et al., 1989). The protein encoded by the third segment is named P3 (instead of PA as in the case of influenza A virus) since it does not contain negative charges at neutral pH. The fourth segment encodes the glycoprotein HEF, the only spike of the viral membrane (Herrler et al., 1988a). The fifth segment encodes the nucleoprotein NP that associates with the viral genome segments along its whole length and builds, together with the polymerases the viral ribonucleoprotein complex (vRNPs) (Nakada et al., 1984b). The sixth segment encodes two proteins, the matrix protein M1, a peripheral membrane protein that covers the viral envelope on its inside, and CM2, a short transmembrane protein supposed to exhibit proton-channel activity required for dissociation of ribonucleoprotein (RNP) complexes from M1 and thus release of uncoated RNPs into the cytoplasm where they are imported into the nucleus to start viral replication. M1 and CM2 are generated by alternative splicing, but in a different manner as described for influenza A virus. Whereas in influenza A virus M1 is translated from a unspliced mRNA, M1 of influenza C virus is generated from a spliced mRNA. Removal of an intron generates the stop codon UGA such that a protein containing 242 residues is translated (Yamashita et al., 1988). The unspliced mRNA encoding CM2 translates into a long precursor protein (374 residues), named p42. P42 contains an internal signal peptide (residues 239–259) which co-translationally targets the protein from the cytosol to the ER and presents it to the translocon. Here residues C-terminal to the signal peptide are translocated into the lumen of the ER until translocation is stopped by a second hydrophobic region (residues 285–308) that functions as the transmembrane region (TMR) of CM2. The signal peptide is then cleaved by signal peptidase yielding the CM2 protein (115 residues) and the p31 protein (259 residues). P31, which is identical in sequence to M1 (except the 18 C-terminal amino acids), is rapidly degraded after cleavage from p42 suggesting that it does not plays any functional role for the viral life cycle (Hongo et al., 1999; Pekosz and Lamb, 1998). Whether CM2 is a proton channel has not been directly demonstrated by biophysical assays, but it alters the intracellular pH in transfected cells and its transmembrane domain can substitute for that of the influenza A virus M2 protein (Stewart and Pekosz, 2012). The seventh vRNA encodes the two non-structural proteins NS1 and NS2 that are also generated via mRNA splicing (Nakada et al., 1985; Nakada et al., 1986). The unspliced mRNA is translated into the NS1 protein (246 residues) and the spliced mRNA translates the shorter NS2 protein (182 residues). The N-terminal 62 residues of NS1 and NS2 are identical in sequence, splicing then generates a shift in the ORF such that the remaining residues are translated from a different reading frame (Alamgir et al., 2000).

All ORFs are flanked by non-coding (NC) sequences, which are more variable in length than those of influenza A and B virus (Crescenzo-Chaigne et al., 2008). Non-coding sequences are divided into conserved and non-conserved sequences. The first twelve nucleotides at each 3′ end (3′-UCGUU/CUUCGUCC-5′) as well as the last eleven nucleotides at each 5′ end (5′-AGCAGUAGCAA-3′) are conserved between genome segments (Desselberger et al., 1980; Robertson, 1979) and are partially complementary to each other, which enables the single stranded RNA to form a “panhandle” structure (Cheong et al., 1999; Desselberger et al., 1980). This peculiar structure serves as the promotor for transcription of cRNAs and vRNAs, and is required for the endonuclease activity of the viral polymerase complex (Crescenzo-Chaigne et al., 2008; Fodor et al., 1994; Hsu et al., 1987). A uridine-rich region located at position 17 to 22 at the 5′ end of each segment is the template for the poly A tail present at the 3′ end of each mRNA (Desselberger et al., 1980).

## HEF PROTEIN OF INFLUENZA C VIRUS

While influenza A and B virus contain the two glycoproteins Hemagglutinin (HA) and Neuraminidase (NA) inserted into the viral membrane, influenza C virus possesses only one spike designated Hemagglutinin-Esterase-Fusion (HEF) protein which combines the functions of both HA and NA (Herrler et al., 1988a; Herrler and Klenk, 1991). Like HA, it recognizes and binds to a receptor on the cell surface to initiate virus entry. However, the receptor determinant is not N-acetyl-neuraminic acid, but another derivative of neuraminic acid, namely N-acetyl-9-O-acetylneuraminic acid (Rogers et al., 1986). HEF also catalyzes fusion of the viral envelope with endocytic vesicles by a mechanism that is believed to be similar to the well characterized fusion activity of HA. Finally, HEF is the receptor-destroying enzyme, which is the function of the neuraminidase (NA) in influenza A and B virus. HEF does not cleave the terminal sialic acid residue from carbohydrates, but has an esterase activity that removes the acetyl group from position C-9 of N-acetyl-9-O-acetylneuraminic acid (Herrler et al., 1985). This function is probably required to release freshly budded virus particles from infected cells, which would otherwise be trapped at their plasma membrane if the receptor would still be present. Interestingly, HEF can substitute for both HA and NA to support influenza A virus replication if its gene is equipped with the packaging signals from influenza A virus (Gao et al., 2008). After a description of the structure and the modifications of HEF its three functional activities will be discussed in more detail below.

## PRIMARY STRUCTURE OF THE HEF PROTEIN

All full-length HEF protein sequences present in the influenza virus database (http://www.ncbi.nlm.nih.gov/genomes/FLU/Database/nph-select.cgi) contain 641 amino acids (aa, excluding signal peptide), except HEF from one strain which is one amino acid shorter. HEF (like HA) is a typical type 1 transmembrane protein with a short N-terminal, cleavable signal peptide (14 amino acids), a long ectodomain (612 aa), a transmembrane region (26 aa) and a very short cytoplasmic tail (three aa). HEF present in infectious virus particles is composed of two subunits, the N-terminal 432 amino acids are the HEF1 polypeptide, the remaining sequence including the hydrophobic fusion peptide, the transmembrane domain (TMD) and the cytoplasmic tail is called HEF2 (Nakada et al., 1984a; Pfeifer and Compans, 1984). HEF proteins of the novel influenza C-like viruses (influenza D virus) contain a very similar number of amino acids (664 including signal peptide) as HEF from influenza C virus, are predicted to also adopt a type I membrane topology, but the amino acid identity with HEF is only ~53% (Hause et al., 2013).

According to a phylogenetic analysis of their HEF genes the existing strains are divided into six genetic and antigenic lineages, Taylor/1233/47, Aichi/1/81, Sao Paulo/378/82, Kanagawa/1/76, Yamagata/26/81 and Mississippi/80 (Matsuzaki et al., 2003; Muraki and Hongo, 2010; Muraki et al., 1996; Speranskaia et al., 2012). However, there is very little sequence variation (Table S1) and thus influenza C virus was considered to be monosubtypic and stable in evolution, but reassortment between strains within the influenza C genus occur frequently which leads to the appearance of new strains better adapted to their host (Matsuzaki et al., 2003; Peng et al., 1994).

We aligned the HEF sequences from the six influenza C virus lineages to reveal amino acid identity and other common characteristics (Table S1). All residues important for the structure of HEF, such as glycosylation sites, cysteine residues (with one exception, Cys 332 in the Taylor lineage, numbering of HEF excluding the signal peptide), the N-terminal region of HEF2 containing the hydrophobic fusion peptide and the amino acids of the receptor-binding and receptor destroying domain of HEF are invariant. In general (and in contrast to the highly variable HA proteins of influenza A and B virus), only a few amino acid residues are not conserved through all lineages of HEF. 35 of them are located in HEF1 and seven in the smaller subunit HEF2. There are three small regions in HEF1 where many of the variable amino acids are clustered; residues 61–65 contain four amino acid substitutions, residue 165–172 six exchanges and residues 190–195 five substitutions (Table S1). In the crystal structure of HEF the variable regions are located in loops at the surface of the trimer; residue 165–172 and 190–195 near the receptor binding site at the top of the molecule and residues 61–65 near the esterase domain (Fig. 4). These amino acids have been shown to be antibody epitopes that gradually change due to antigenic drift (Matsuzaki et al., 1992).

## CRYSTAL STRUCTURE OF THE HEF PROTEIN

**Figure 3:**
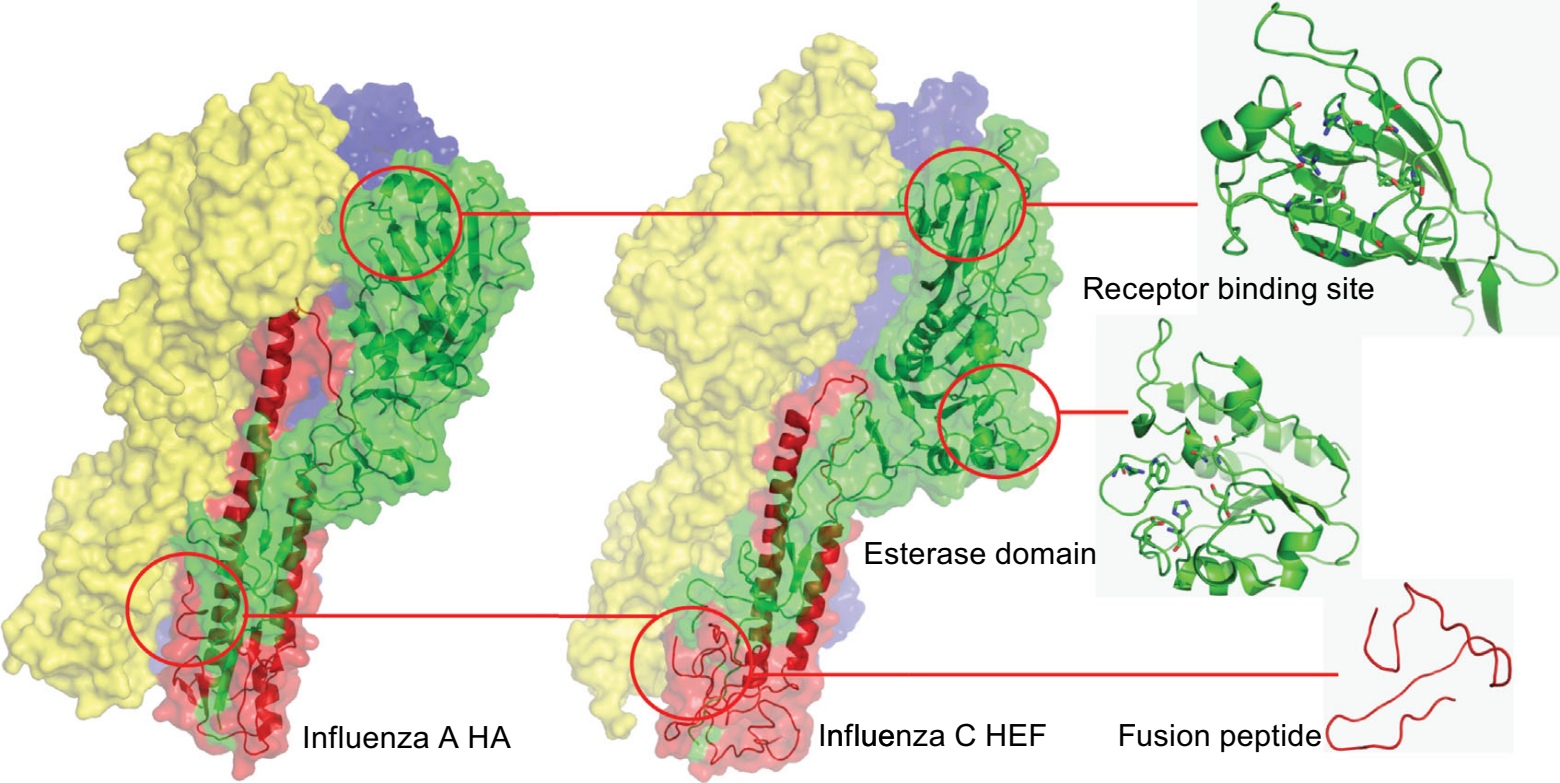
**Comparison of the crystal structure of influenza C virus HEF protein and influenza A virus HA protein**. Only the ectodomains of both proteins were amenable for crystallization. The viral membrane is located beneath the structure. Individual subunits of the trimeric spike proteins are drawn in different colors. The overall structure of both proteins is similar except an additional bulge in the head domain of HEF that contains the esterase activity which is not present in HA. The receptor binding region, esterase domain and the fusion peptide of HEF are enlarged. Figures were created with PyMol from PDB files 1RUZ (HA from influenza A virus strain A/South Carolina/1/1918 (H1N1)) and 1FLC (HEF from influenza C virus strain C/Jhb/1/66 strain). HEF1 and HEF2 subunits of one monomer are drawn in red and green, respectively. The other two monomers are labelled in yellow and blue, respectively

Initial studies using electron microscopy showed that the HEF spike forms a mushroom-shaped trimer consisting of a membrane-near stalk and a globular head (Herrler et al., 1981; Hewat et al., 1984). X-ray crystallography of the bromelain-cleaved ectodomain of HEF then revealed the high resolution structure (4.5 Å) of the HEF trimer. Although there is only 12% amino acid identity between HA and HEF, the overall structure of both molecules as well as folds of individual segments are quite similar, except an additional bulge, which is located at the lower part of the globular domain and contains the esterase region that is not present in HA (Fig. 3). Similar to HA, the receptor-binding region is located at the top of the head domain, which consists only of HEF1 residues. The stalk is formed by three 60 Å long α-helices that contain the whole HEF2 sequence and N-terminal residues 1–40 and C-terminal residues 367–432 of HEF1. The fusion peptide at the N-terminus of HEF2 is located around 35 Å above the membrane, but in contrast to HA, the first four residues are exposed at its surface and not buried within the trimer (Rosenthal et al., 1998; Zhang et al., 1999). The detailed structure of the receptor binding site and the catalytic center of the esterase activity will be discussed in more detail in the last paragraph.

## CO- AND POST-TRANSLATIONAL MODIFICATIONS OF HEF

HEF is synthesized on membrane-bound ribosomes and the primary translation product is subjected to a series of co- and post-translational modifications, most of them are required for proper folding and/or functioning. Already during translocation of HEF into the lumen of the ER the N-terminal signal peptide is cleaved, carbohydrates are attached and intramolecular disulfide linkages are formed and probably remodeled. These co-translational modifications affect folding of the molecule and its trimerization, processes which are (at least in HA and other viral glycoproteins) a prerequisite for exit of cargo from the ER (Doms et al., 1993). Later on a long chain fatty acid is attached to a cysteine located at the end of the transmembrane region and HEF is proteolytically cleaved into the subunits HEF1 and HEF2, a process that is essential for virus replication. Timing of both modifications has not been analyzed for HEF, but in HA palmitoylation occurs prior to proteolytic cleavage (Veit and Schmidt, 1993).

## LOCATION OF N-GLYCOSYLATION SITES IN THE CRYSTAL STRUCTURE OF HEF

**Figure 4:**
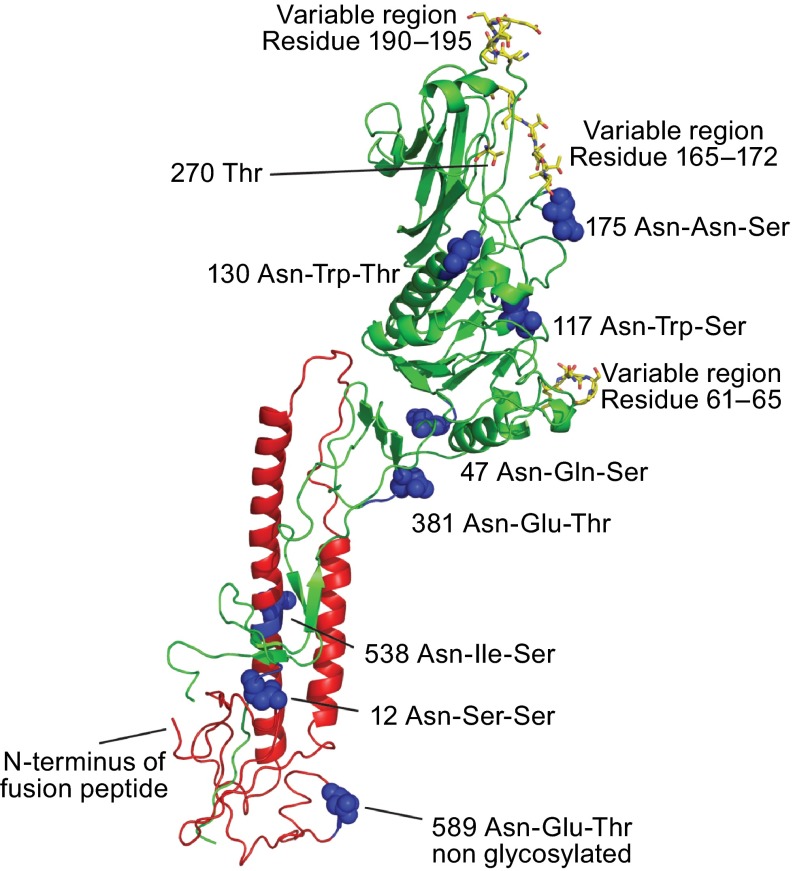
**Location of N-Glycosylation sites and variable regions in the crystal structure of HEF**. Asparagine residues of used and unused glycosylation sequons (Asn-X-Ser/Thr) are depicted in the secondary structure of a HEF monomer as highlighted as balls. Amino acids in the HEF protein from C/JHB/1/66, which vary in other influenza C virus isolates are marked as sticks (Table S1). Threonine residue 270, which is exchanged by isoleucine in a virus variant that has acquired the ability to grow in MDCKII cells, is also marked as sticks. (This residues is termed Thr284 in the publication (Szepanski et al., 1992) since the 14 amino acid long signal peptide was included in the numbering). Figure was created with PyMol from PDB file 1FLC. HEF1 and HEF2 subunits are drawn in red and green, respectively

HEF, like HA, contains only asparagine-linked carbohydrates; O-glycosylation does not occur (Herrler and Klenk, 1991; Hongo et al., 1986a). The composition of the carbohydrate chains has not been precisely determined, but apparently, some of them are not terminally glycosylated since they are not processed to an Endo-H resistant form (Pekosz and Lamb, 1999). The location of the individual glycosylation sites in the crystal structure of HEF is depicted in Fig. 4. Seven of the eight highly conserved N-glycosylation sequons (Asn-X-Ser/Thr) are used. One is located in HEF2 and six in HEF1, three in the globular head and two in the hinge region that connects the stalk with the head. The site at position 589 is not glycosylated, probably because it is located too close to the membrane-spanning region and cannot be accessed by the oligosaccharide transferase (Herrler and Klenk, 1991; Nakada et al., 1984a; Pfeifer and Compans, 1984) (Fig. 4 and supplementary table). Although the position of carbohydrates attached to HA of influenza A virus changes and their number increases during viral evolution (Skehel and Wiley, 2000), their distribution is quite similar to that of HEF, i.e. the majority is located in the larger subunit. Glycosylation of HEF is crucial for proper folding of the glycoprotein by protecting it from proteolytic degradation and hence important for the presentation of antigenic epitopes (Hongo et al., 1986b).

## LOCATION OF INTRAMOLECULAR DISULFIDE BONDS IN THE CRYSTAL STRUCTURE OF HEF

**Figure 5:**
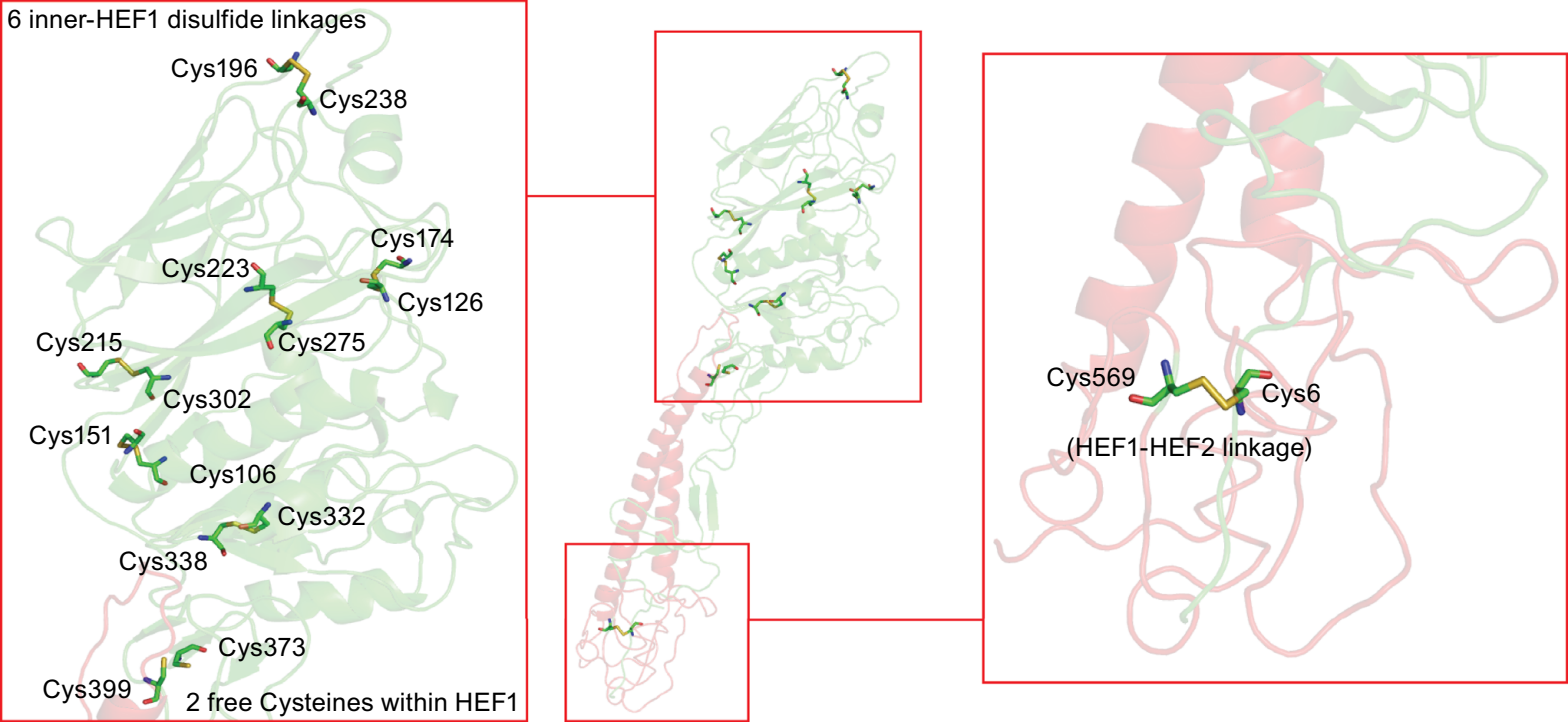
**Location of intramolecular disulfide linkages in HEF**. The middle part shows the secondary structure of a HEF monomer. HEF1 and HEF2 subunits are drawn in red and green, respectively. The left part shows the head domain which contains six disulfide linkages and also two free cysteines. The right part shows the location of the only disulfide linkage between HEF1 and HEF2. The figure was created with PyMol from PDB file 1FLC. HEF1 and HEF2 subunits are drawn in red and green, respectively

There are 15 cysteine residues in HEF1, twelve of them form six intrachain disulfide linkages that stabilize the globular head domain. Their location is depicted in the crystal structure of HEF in Fig. 5. Cysteine 332 is not required for proper folding and functioning of HEF since it is exchanged by a tyrosine in the Taylor lineage of influenza C virus. Two cysteines, Cys373 and Cys399 that do not form a disulfide linkage in the mature protein are located at the hinge that connects the globular head with the stalk region. The remaining cysteine in HEF1 forms an interchain disulfide bond with the only cysteine residue in the ectodomain of HEF2, which is located at the bottom of the trimer. A similar distribution of disulfide bonds is present in HA of influenza A virus, i.e. one disulfide bond connects HA1 with HA2; the majority are intrachain bonds, three or four in HA1 and just one in HA2 (Segal et al., 1992; Skehel and Wiley, 2000). The rare occurrence of disulfide-bonds in HEF2 and HA2 allows this subunit to perform the large conformational changes that catalyze membrane fusion.

## FOLDING AND INTRACELLULAR TRANSPORT OF HEF

HEF (at least from the strain C/JHB/1/66) is subject of a complicated folding procedure involving the formation and remodeling of intramolecular disulfide bond (Szepanski et al., 1994). In virus-infected cells freshly synthesized HEF has an apparent molecular weight (MW) of 80 kDa as demonstrated by both reducing and non-reducing SDS-PAGE. This corresponds to the predicted molecular weight of the glycosylated form of the protein (Sugawara et al., 1981). Subsequently HEF is converted to a form with a MW of 100 kDa, which appears after non-reducing, but not after reducing SDS-PAGE, indicating that intramolecular disulfide-bond formation causes the decrease in electrophoretic mobility (Herrler et al., 1981). Besides reduction of disulfide bonds, proteolytic cleavage also converts the 100 kDa into the 80 kDa form suggesting that the 100 kDa form possesses a strained conformation (Herrler et al., 1979; Szepanski et al., 1994).

When HEF from C/JHB/1/66 was expressed from cDNA in the absence of the other viral proteins, conversion into the 100 kDa form was either very inefficient or not observed at all suggesting that the interaction of HEF with other viral proteins is required for folding (Szepanski et al., 1994). Expressed HEF is not transported to the cell surface, which is in line with the established paradigm that proper folding is a prerequisite for exit of proteins from the ER and hence transport to the plasma membrane (Doms et al., 1993). The defect in disulfide bond formation and surface transport was partially overcome by either deleting its short cytoplasmic tail (Arg-Thr-Lys), replacing it by the longer cytoplasmic tail of influenza A virus HA or exchanging the two basic amino acids to acidic or hydrophobic residues (Oeffner et al., 1999; Szepanski et al., 1994).

In contrast, HEF proteins from the strains C/California/78, C/Ann Arbor/1/50 and C/Taylor/1233/47 were efficiently transported to the plasma membrane in the absence of other viral proteins (Pekosz and Lamb, 1999; Vlasak et al., 1987). In addition, conversion of an 80 kDa to a 100 kDa band was not obvious by SDS-PAGE, although heterogeneity of bands after non-reducing SDS-PAGE suggests that remodeling of disulfide bonds also occurs (Pekosz and Lamb, 1999). The reason for this strikingly different behavior of HEF proteins is unknown, but either subtle amino acid differences between HEF proteins of different virus strains or between the cloned HEF-gene and the gene present in virus particles of C/JHB/1/66 have been discussed (Pekosz and Lamb, 1999).

There are also other indications that folding of HEF is more complicated than folding of HA. Whereas HAs from several influenza A virus strains have passed the medial-Golgi (determined as acquisition of Endo-H resistant carbohydrates), around 15 min after synthesis and are rapidly (t_1/2_: 30 min) and completely transported to the cell surface (Engel et al., 2012), intracellular transport of HEF is slow and incomplete. Half times of more than 60 min for acquisition of Endo-H resistant carbohydrates and for transport to the plasma membrane have been reported and only a fraction (70%) of all synthesized molecules appear at the cell surface (Pekosz and Lamb, 1999).

In addition, HEF exhibits intrinsic temperature sensitivity. Expression levels of HEF at the plasma membrane are two times higher at 33°C compared to 37°C and, probably as a consequence, membrane fusion is more efficient at 33°C than at 37°C. Since trimerization of HEF is also reduced at 37°C the underlying cause of reduced cell surface exposure is slower and less efficient folding of HEF at higher temperatures (Takashita et al., 2012) which is reminiscent of temperature sensitive mutants of HA of influenza A virus (Garten et al., 1992).

The temperature sensitivity of HEF is probably an adaption of the virus to replicate only in the upper respiratory tract that has, due to contact with inhaled air before it is warmed up, a lower temperature than the lower respiratory tract. In the lab (cell culture and chicken embryos) influenza C virus is also amplified at 33°C where it grows to higher titers than at 37°C (Crescenzo-Chaigne and van der Werf, 2007; O’Callaghan et al., 1977; Pachler et al., 2010; Wagaman et al., 1989). However, other proteins also influence the temperature preference for virus replication since the polymerase also exhibits a higher activity at 33°C than at 37°C (Nagele and Meier-Ewert, 1984).

## S-ACYLATION AND RAFT-LOCALIZATION OF HEF

**Figure 6:**
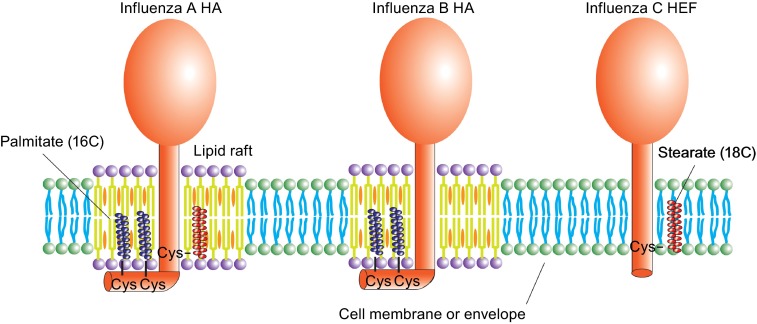
**Different acylation and lipid raft association of HA and HEF protein**. HAs of influenza A virus contain one stearate attached to a cysteine positioned at the end of the transmembrane region and two palmitates attached to cytoplasmic cysteines. HA of influenza B virus possesses two palmitates attached to cytoplasmic cysteines. HEF of influenza C virus has only one stearate attached to a transmembrane cysteine. Whereas HA of influenza A and B virus are associated with membrane rafts, cholesterol- and sphingolipid-enriched nanodomains of the plasma membrane, HEF is thought to localize to the bulk phase of the plasma membrane

Another common modification of viral glycoproteins is the covalent attachment of fatty acids, usually palmitate (C 16:0) in a thioester-type linkage to cysteine residues located either at the cytosol-facing end of the transmembrane region or in the cytoplasmic tail (Veit, 2012; Veit et al., 2013). HEF of influenza C virus is unique in this aspect, since it contains mainly stearic acid linked to cysteine 652 (Veit et al., 1990; Veit et al., 1996) (Fig. 6). This longer chain fatty acid (C 18:0) was initially identified by chromatographic determination of HEF-bound, [^3^H]-labelled fatty acids, but results were recently confirmed by mass-spectrometry with C-terminal anchoring fragments of HEF purified from virus particles (Kordyukova et al., 2008). These studies revealed also that influenza B virus HA possessing two cytoplasmic cysteines contains only palmitate, whereas HAs of influenza A virus having one transmembrane and two cytoplasmic cysteines contain both palmitate and stearate, but the latter is exclusively attached to the cysteine positioned in the transmembrane region (Kordyukova et al., 2008; Naeve and Williams, 1990; Naim et al., 1992; Steinhauer et al., 1991; Veit et al., 1990; Veit et al., 1991) (Fig. 6). It was originally proposed that the different length of the cytoplasmic tails of HA (11 aa) and HEF (3 aa) could be the reason for different fatty acid selection (Veit et al., 1996), but a recent comprehensive mutagenesis study with HA revealed that the location of a cysteine relative to the transmembrane region is the decisive factor for selective attachment of stearate (Brett et al., 2014). Enzymes that attach palmitate and stearate to HA or HEF (or to other viral glycoproteins) have not been identified so far, but likely candidates are members of the family of DHHC-proteins, polytopic membrane proteins with the Glu-His-His-Cys motif in one of their cytoplasmic loops, that are known to acylate cellular proteins (Greaves and Chamberlain, 2011).

Acylation of HA of influenza A virus is essential for virus replication, since (depending on the virus strain) either virus mutants with more than one acylation site deleted show drastically impaired growth or could not be created at all by reverse genetics (Chen et al., 2005; Wagner et al., 2005; Zurcher et al., 1994). Recombinant virus lacking the acylation site of HEF could be rescued, but viral titers were reduced by one log relative to wild type Flu C (our unpublished results). The resulting virus particles have a regular protein composition and no changes in their morphology were obvious by electron microscopy, but their hemolytic activity is reduced indicating a defect in membrane fusion. This is in accordance with results on several HA subtypes showing that (i) the stearoylated cysteine at the end of the transmembrane span is less important for virus replication compared to the two cytoplasmic palmitoylated cysteines and that (ii) acylation affects opening of a fusion pore (Sakai et al., 2002; Ujike et al., 2004; Wagner et al., 2005). However, for H2 subtype HA it was reported that acylation does not influence HA’s membrane fusion activity, but plays an essential role for virus particle assembly (Chen et al., 2005; Zurcher et al., 1994).

Several studies illuminated the essential role of palmitoylation for association of HA with rafts, cholesterol and sphingolipid-enriched nanodomains on the cellular plasma membrane that serves as the viral assembly and budding site (Engel et al., 2010; Levental et al., 2010; Melkonian et al., 1999; Veit and Thaa, 2011) (Fig. 6). Interestingly, HEF is apparently not a component of rafts, at least it does not associate with detergent-resistant membranes, their controversial biochemical correlate indicating that virus particles buds from the bulk phase of the plasma membrane (Zhang et al., 2000).

## PROTEOLYTIC CLEAVAGE OF HEF

After synthesis of the precursor HEF0 a yet unknown protease hydrolyses the peptide bond between Arg432 and Ile433, which is located in the stem region of the trimeric spike and thus in a similar position as the cleavage site in HA (Klenk et al., 1975; Lazarowitz and Choppin, 1975; Rosenthal et al., 1998; Sugawara et al., 1981). The resulting subunits HEF1 and HEF2 remain covalently connected by a single disulfide linkage, which is also located in the stalk region of the protein. Proteolytic cleavage is an essential prerequisite for the membrane fusion activity of HEF (and also of HA) since it enables the protein to get activated by low pH (Herrler et al., 1981; Kitame et al., 1982; Ohuchi et al., 1982).

HEF proteins from all influenza C virus strains contain a monobasic cleavage site and are in this respect similar to HAs from human, porcine, equine and low pathogenic avian influenza A viruses (Herrler and Klenk, 1991; Neumann and Kawaoka, 2006). Polybasic cleavage sites that are present in HA of highly pathogenic avian influenza A viruses and processed by the ubiquitous protease furin are not found in any HEF protein. Consequently, replication of influenza C virus is limited to the site of virus infection, the respiratory tract. Spread to other tissues or even systemic infection, as observed for highly pathogenic avian influenza virus having a multibasic cleavage site between HA1 and HA2, does not occur with influenza C virus (Horimoto and Kawaoka, 1994; Stieneke-Grober et al., 1992). Multiple replication cycles of influenza C virus in tissue culture are enabled by addition of trypsin, whereas embryonated eggs produce infectious virus with cleaved HEF (Herrler et al., 1979; Sugawara et al., 1981).

The enzyme catalyzing proteolytic cleavage of HEF has not been identified so far, but since both HA and HEF can be cleaved by trypsin at similar concentrations *in vitro* (5~20 µg/mL) it seems likely that they are also activated by the same (or very similar) enzymes inside cells (Herrler et al., 1979; Nerome et al., 1979). Likely candidates are members of the family of type II transmembrane serine proteases, such as HAT (human airway trypsin-like protease), TMPRSS2 and TMPRSS4 (transmembrane protease serine S1, members 2 and 4). These enzymes are expressed at the plasma membrane of human bronchial/tracheal epithelial cells where they cleave HA from various, but not all subtypes (Bottcher-Friebertshauser et al., 2013).

## REGULAR ARRANGEMENT OF HEF SPIKES IN VIRUS PARTICLES

Electron microscopy revealed another unique feature of influenza C virus particles not observed for influenza A and B virions. HEF trimers on the surfaces of both spherical and filamentous particles are arranged in a reticular structure that has been described to consist mainly of hexagons (Compans et al., 1977; Flewett and Apostolov, 1967; Waterson et al., 1963). The regular polymeric reticular structure can be observed not only on the surface of intact viral particles, but also when HEF is removed from the membrane, either by limited proteolytic digestion or by spontaneous release (Herrler et al., 1981). These results indicate (i) that the hexagonal arrangement is an intrinsic feature of HEF and does not require other viral proteins such as M1 and (ii) its formation likely involves lateral interaction between the ectodomains of HEF; the TMR and cytoplasmic tail are not required to maintain the structures. Which amino acids form lateral interactions between HEF trimers and which function it serves for virus replication has not been investigated. One might speculate that the formation of a regular arrangement of HEF trimers on the plasma membrane might induce membrane curvature, i.e. it acts like an extrinsic coat that might help to sculpt a virus particle out of the membrane. However, the lateral arrangement of exclusively hexagons would result in the formation of a flat structure without any curvature. Thus, in order to create and cover a spherical particle, HEF must form a precisely defined arrangement of pentagons and hexagons.

Virus budding might be reinforced by the matrix protein M1 that has been shown to form virus-like particles when expressed in the absence of other viral proteins (Muraki et al., 2004). M1 might execute a pushing force by oligomerization at the inner site of the plasma membrane. How these two assumed activities of HEF and M1 are coupled is not obvious since the cytoplasmic tail of HEF is very short, only three amino acids, and might thus not be able to bind to M1 with high affinity.

## RECEPTOR BINDING ACTIVITY OF HEF

**Figure 7:**
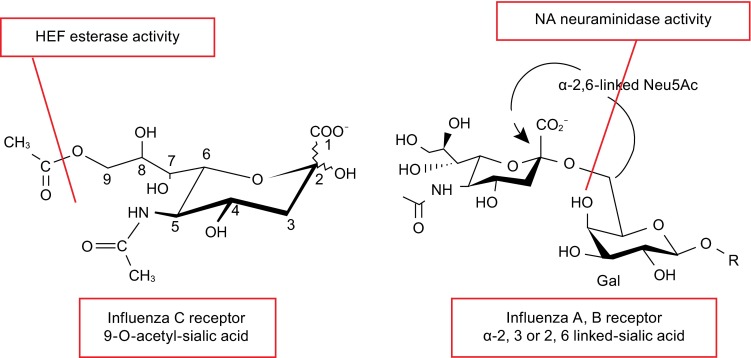
**Cellular receptors and receptor-destroying activity of influenza C virus and influenza A and B virus**. The structure of cellular receptors for HEF from influenza C virus (N-acetyl-9-O-acetylneuraminic acid) and HA from influenza A and B virus (N-acetylneuraminic acid) are shown. Both neuraminic acid derivatives are the terminal sugars in carbohydrate chains attached to glycolipids or glycoproteins located at the cellular surface. Subtypes of influenza A virus HA discriminate between an α2-6 and α2-3 linkage to the second galactosyl residue, a property that (partially) explains species specificity. HEF of influenza C virus apparently recognizes N-acetyl-9-O-acetylneuraminic acid independent of its linkage to the next sugar. HEF has also esterase activity that cleaves acetyl from the C9 position. In influenza A and B virus the receptor-destroying activity is performed by the NA protein, which hydrolyzes the glycosidic bond between sialic acid and galactosyl residues. The cleaved bonds are indicated by a red line

HA of influenza A and B virus and HEF of influenza C virus use different neuraminic acid derivatives as receptor (Fig. 7). HEF binds to N-acetyl-9-O-acetylneuraminic acid (9-O-Ac-Neu5Ac), which can be present on both glycolipids and glycoproteins to function as viral receptor (Herrler et al., 1988a; Herrler and Klenk, 1987; Herrler et al., 1987; Rogers et al., 1986). Likewise, HEF binds to its receptor regardless of whether 9-O-Ac-Neu5Ac is attached via an α-2,3 or α-2,6 linkage to the following galactosyl residue (Rogers et al., 1986). In contrast, HA uses terminal N-acetylneuraminic acid (Neu5Ac) and the glycosidic bond of Neu5Ac influence the host specificity. Avian influenza viruses usually bind to Neu5Ac-α2,3-Gal while mammalian influenza viruses usually bind to Neu5Ac-α2,6-Gal (Thomas and Noppenberger, 2007; Trebbien et al., 2011). The unique receptor specificity of influenza C virus has been used as an efficient tool to detect 9-O-Ac-Neu5Ac on the surface of various cells (Martin et al., 2003; Muchmore and Varki, 1987; Zimmer et al., 1994).

There is some evidence that the abundance of 9-O-Ac-Neu5Ac in cultured cells influences the tropism of influenza C virus. Influenza C virus is usually grown in MDCK I cells, whereas another subline of Madin-Darby canine kidney cell, MDCK II cells are (due to insufficient number of receptors) resistant against virus infection. A mutant of influenza C virus with the ability to replicate in MDCK II cells has an amino acid exchange from threonine to isoleucine at position 270 (see Fig. 4 for the location of Thr270 in the crystal structure of HEF) that apparently increases the affinity of HEF for its receptor (Szepanski et al., 1992). Using reverse genetics it was recently confirmed that the exchange from threonine to isoleucine is necessary and sufficient to enable influenza C virus to grow in MDCK II cells (Crescenzo-Chaigne and van der Werf, 2007).

**Figure 8:**
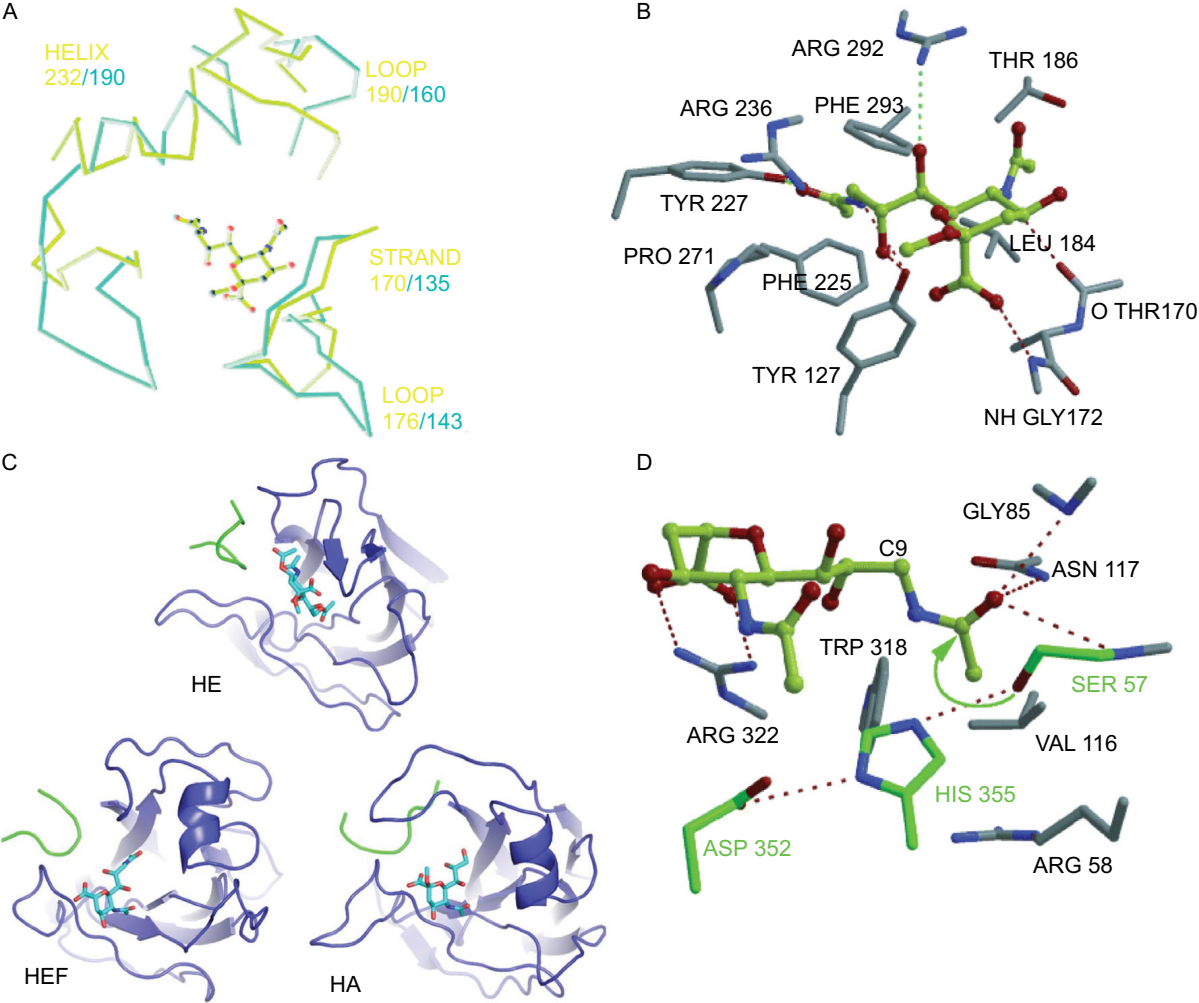
**Structures of receptor binding site and esterase site of HEF, HA and HE**. (A) Superimposing of HEF and HA receptor binding sites complexed with ligand (9-acetamidosialicacid α-methyl glycoside). The yellow-green lines and the light blue lines represent the binding elements of HA and HEF, respectively. (B) Structure of HEF binding sites complexed with the receptor. Key residues forming hydrogen bonds with the ligand are shown. (C) Comparison of the receptor binding topology of the hemagglutinin-esterase (HE) protein of coronavirus, HEF and HA with their ligands. The bound ligands are αNeu4,5,9Ac_3_2Me in HE, αNeu5,9Ac_2_2Me in HEF and αNeu5Ac2Me in HA are shown in stick representation. The ligand is bound to HE in an opposite orientation compared to HEF and HA. (D) Structure of the esterase active site of HEF. Key residues forming hydrogen bonds with the ligand are shown. (A), (B) and (D) were taken from reference (Rosenthal et al., 1998) and (C) from reference (Zeng et al., 2008) with permission

The crystal structure shows that HEF binds to 9-O-Ac-Neu5Ac in a similar pattern as HA binds to Neu5Ac. The binding elements consist of an α-helix, a loop and an extended strand (Fig. 8A). The key residues for binding HEF to 9-O-Ac-Neu5Ac are shown in Fig. 8B. Tyr127, Thr170, Gly172, Tyr227 and Arg292 form hydrogen bonds with hydroxyl-groups of the ligand, and some other residues form the structural support of the receptor binding site. The HEF binding site also contains a unique hydrophobic pocket that accommodates the acetyl methyl group (Rosenthal et al., 1998).

Some coronaviruses, such as the prototype member mouse hepatitis virus (MHV) and human and bovine coronavirus, contain a hemagglutinin esterase (HE) protein that also uses 9-O-Ac-Neu5Ac as receptor (Mayr et al., 2008; Schwegmann-Wessels and Herrler, 2006; Vlasak et al., 1988). The crystal structure of HE from bovine coronavirus revealed that the ligand is bound in an opposite orientation compared to HEF and HA (Zeng et al., 2008) (Fig. 8C).

## RECEPTOR HYDROLYSIS (ESTERASE) ACTIVITY OF HEF

In accordance with its receptor binding specificity, HEF is an esterase that cleaves acetyl from the C9 position of terminal 9-O-Ac-Neu5Ac residues to release virus particles from infected cells (Herrler et al., 1988a; Herrler et al., 1985; Mayr et al., 2008) (Fig. 7). The esterase activity of HEF belongs to the class of serine hydrolase, where the −OH group of a serine residue performs a nucleophilic attack on the carbonyl-group of the substrate. Since the −OH group is not sufficiently nucleophilic it is activated by two other amino acids that together build the typical catalytic triad of serine hydrolases, the amino acids serine, histidine and aspartic acid. The base histidine polarizes and deprotonates the −OH-group of serine to increase its reactivity whereas aspartic acid aligns and polarizes the histidine (charge relay system) (Herrler et al., 1988b; Kraut, 1977; Pleschka et al., 1995).

Crystallography in the presence of two non-hydrolysable receptor analogues of HEF revealed that serine 57, aspartic acid 352 and histidine 355 are the key residues for the acetylesterase activity of HEF (Rosenthal et al., 1998) (Fig. 8D). Prior to that it has already been shown that mutation of Ser57 and His355 completely abolished the enzymatic activity of HEF, essentially confirming the data from crystallography, but mutation of other residues in the vicinity, i.e. Asp247, Asn266 and His354 also affected the hydrolytic activity of HEF (Pleschka et al., 1995).

Ser57 is positioned for nucleophilic attack on the carbonyl carbon of the 9-O-Ac-Neu5Ac group. The carbonyl oxygen of the substrate points into an ‘oxyanion hole’ formed by the side chain of Asn117 and the NH -groups of Gly85 and Ser57 (Rosenthal et al., 1998). Arg 322 of HEF forms two hydrogen bonds with the sialoside carboxylate group (Fig. 8D). The structure of the esterase site is quite similar between HEF and coronavirus HE. The catalytic triad of HE consists of the same amino acids, i.e. Ser40, His329 and Asp326; Ser40 also forms an oxyanion hole with the side chains of Gly75 and the NH group of Asn104 (Zeng et al., 2008).

## MEMBRANE FUSION ACTIVITY OF HEF

Membrane fusion between the viral envelope and endocytic vesicles is the crucial step to release the viral genome into the cytoplasm of the cell (Hamilton et al., 2012; Skehel and Wiley, 2000). There are two essential requirements for both HEF and HA to catalyze membrane fusion: (i) The precursor proteins HEF0 and HA0, must be cleaved into the subunits HEF1 (HA1) and HEF2 (HA2). (ii) The proteins must then be exposed to acidic pH to become fusogenic. This was initially demonstrated for influenza viruses by a simple membrane fusion assay, hemolysis of erythrocytes that occurs only if virus particles containing cleaved HA or HEF are exposed to acidic pH (Formanowski et al., 1990; Huang et al., 1981; Kitame et al., 1982; Lenard and Miller, 1981; Maeda and Ohnishi, 1980; Ohuchi et al., 1982). Biochemical assays subsequently revealed that low pH initiates a conformational change since molecules become susceptible to proteolytic digestion (Formanowski et al., 1990). The low pH is thought to cause protonation of specific amino acids that triggers the following large scale rearrangement of the proteins. Histidines might play this role since their pKas match the pH of endosomes (5.5–6). For HA of influenza A virus specific histidine residues have been identified (Mair et al., 2014), but similar studies have not been performed with HEF. In both influenza A and C virus threshold pH values that initiate membrane fusion differ from strain to strain by about 0.7 pH units. This does not necessarily mean that different histidines are the relevant target of protonation, but that (between strains) variable amino acids in the vicinity of a specific histidine affect its pKa. For influenza C virus pH values required to cause hemifusion (measured as lipid mixing, range of 5.6–6.1) are 0.3–0.6 pH units higher than pH values for full fusion (measured by hemolysis, range of 5.1–5.7) (Formanowski et al., 1990).

Interestingly, kinetic studies with influenza C virus revealed a lag phase before onset of fusion that is not observed with influenza A and B virus (Formanowski et al., 1990). It is likely that the lag phase reflects dispersion of the lateral arrangement of HEF spikes on the viral membrane that might hinder HEF’s conformational change. Accordingly, when virus particles are treated with low pH before electron microscopy, HEF spikes are less well ordered and the typical hexagonal structure disappeared (Hewat et al., 1984).

**Figure 9:**
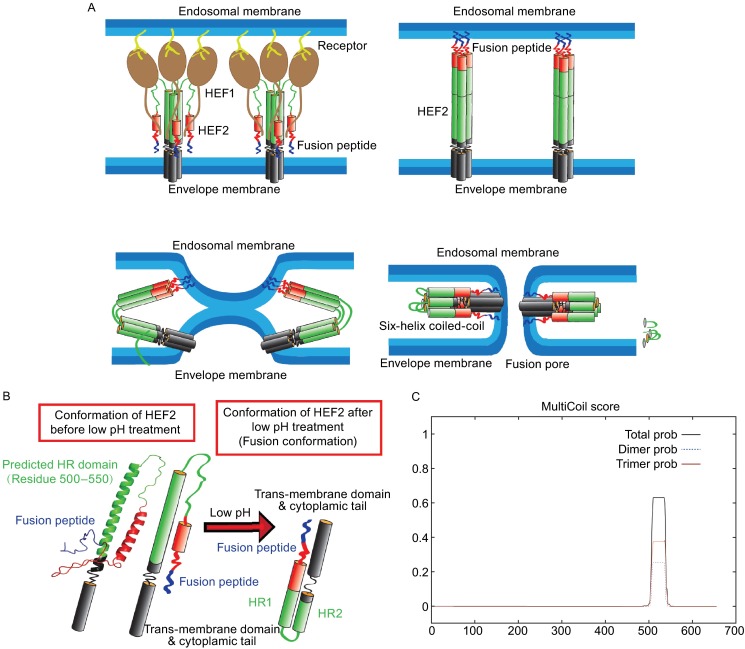
**Probable mechanism for HEF-mediated membrane fusion**. (A) Probable conformational change of HEF2 during membrane fusion. Left part: structure of the HEF2 subunit at neutral pH. The HR is located in the long α-helix of HEF2 and thus in a similar position as the HR in HA. Right part: hypothetical structure of the HEF2 subunit at acidic pH. (B) Hypothetical scheme for the HEF-catalyzed membrane fusion mechanism. Upper left part: HEF binds to its receptor via its HEF1 subunit (brown) and is endocytosed. Upper right part: Acidification of the endosome causes a conformational shift in HEF2. The fusion peptide, which was (partially) buried in the stalk is exposed and inserts into the endosomal membrane. Lower, left part: The middle part of the HR domain (green) changes its conformation from a helix to a loop, which causes bending of the molecule and close apposition of viral and cellular membrane allowing the exchange of lipids (hemifusion). Lower, right part: Interactions between the fusion peptide and the TMD of HEF might cause opening of a fusion pore. (C) Prediction of a heptad repeat (HR) in HEF by online software “multicoils scoring form”

The molecular details of the subsequent refolding of HEF have not been revealed, but it is believed that they are similar to the well characterized conformational changes of HA that were elucidated by a comparison of the crystal structure of HA at neutral pH with the structure of a HA fragment after low pH treatment (Bullough et al., 1994). The first conformational change removes the fusion peptide from its buried location at the bottom of the stalk and exposes it at the surface of the molecule such that it can insert into the endosomal membrane (“jackknife mechanism”). A second conformational change then bends the ectodomain thereby drawing the fusion peptide towards the transmembrane region. This leads to a close apposition of viral and endosomal membranes, hemifusion with exchange of lipids, opening of a fusion pore and eventually complete merger of both lipid bilayers. The second conformational change requires so-called “heptad repeats”, amphipathic helices which interact to form a stable 6-helix coiled coil domain (Cross et al., 2009; Harrison, 2008; Kemble et al., 1994; Skehel and Wiley, 2000) (Fig. 9A and 9B). With the software “Multicoil Scoring Form” (http://groups.csail.mit.edu/cb/multicoil/cgi-bin/multicoil.cgi), a highly probable heptad repeats domain was found between amino acids 500 and 540, residues that encompass the long α-helix of HEF2. They are thus in a similar position as the heptad repeats that form the six-helix bundle coiled-coil in the low pH structure of HA (Fig. 9C). This region of HEF thus might convert from a long, uninterrupted helix into two smaller, antiparallel helices, which are connected by a loop and form the coiled-coil domain that stabilizes the fusion conformation (Bullough et al., 1994) (Fig. 9A and 9B).

The fusion peptides of HA and HEF have similar, but also different features. The first 23 amino acids of HA2 (GLFGAIAGFI**E**GGWTGMI**D**GWYG, sequence of H1 subtype) is highly conserved between subtypes and contains hydrophobic, aromatic, but also some negatively charged residues. It is also characterized by GxxG and GxxxG motifs that are known to mediate interactions between transmembrane segments. In a lipid environment the fusion peptide of HA forms a boomerang-like structure (aa 1–20) or a (tighter) helical hairpin (aa 1–23) (Han et al., 2001; Lorieau et al., 2012). The sequence at the N-terminus of HEF2 (IFGI**DD**LIIGLLFVAIV**E**AGIGG) is not conserved to that of HA2. However, if the first six residues, which are not buried within the trimeric stalk, are not taken into account some sequence homology between HA and HEF is apparent. The fusion peptide of HEF has a similar amino acid composition as that of HA, but glycine residues do not form GxxG or GxxxG motifs. The structure of HEF’s fusion peptide in lipid micelles is not known, but in the HEF trimer it already adopts a loop-like structure.

## WHY STUDY INFLUENZA C VIRUS?

Although influenza C virus is currently not a serious threat to humans, it might be nevertheless fruitful and revealing to study its biology. Whereas the receptor-binding and receptor-destroying activities of HEF are now well characterized and its fusion activity is likely to be similar to that of HA, the mechanism of virus assembly and budding is largely unexplored and might be different for influenza A and C virus. If it is confirmed by more sophisticated methods that HEF does not associate with membrane rafts (Zhang et al., 2000), it is likely that influenza A and C virus bud at different sites of the apical plasma membrane, membrane rafts in the case of influenza A virus (Gerl et al., 2012; Rossman and Lamb, 2011) and the bulk phase or other domains in the case of influenza C virus. Since rafts are believed to enrich viral proteins and deplete many cellular proteins they represent the first concentration step in the assembly of a virus particle that contains very little cellular proteins (Veit and Thaa, 2011). One might speculate that the regular arrangement of HEF trimers might substitute for the concentration of HA in rafts, i.e. its formation might displace cellular proteins from the viral assembly site. A regular arrangement of hexagons and pentagons might then help to shape a virus particle out of the plasma membrane.

For influenza A virus it has been demonstrated that virus scission is achieved by the M2 protein that is targeted to the edge of the assembly site and inserts an amphiphilic helix into the inner leaflet to induce membrane curvature (Rossman et al., 2010). Whether CM2 plays a similar role for release of influenza C virus has not been investigated, but the bioinformatic tool heliquest (http://heliquest.ipmc.cnrs.fr/) predicts the presence of an amphiphilic helix at the beginning of the cytoplasmic tail of CM2. The recent developments of reverse genetics systems for influenza C virus make some of the mentioned questions amenable to experimental verification (Crescenzo-Chaigne and van der Werf, 2007; Muraki et al., 2007; Pachler et al., 2010). Thus, further studies might reveal common and different principles of influenza virus budding that might be helpful to combat the disease.

## Electronic supplementary material

Supplementary material 1 (PDF 620 kb)

## ABBREVIATIONS

9-O-Ac-Neu5Ac: N-acetyl-9-O-acetylneuraminic acid
C, Cys: cysteine
CM2: influenza C ion-channel matrix protein 2
CT: cytoplasmic tail
ER: endoplasmic reticulum
HA_0_: hemagglutinin precursor
HA_1_, HA_2_: hemagglutinin subunits
HEF: hemagglutinin-esterase-fusion protein
HEF_1_, HEF_2_: hemagglutinin-esterase-fusion protein subunits
kDa: kilodalton
MDCK I: Madin- Darby canine kidney cell type I
MDCK II: Madin- Darby canine kidney cell type II
M1: matrix protein 1
NA: neuraminidase
Neu5Ac: N-acetylneuraminic acid
ORF: open reading frame
RNP: ribonucleoprotein complex
S, Ser: serine
SDS-PAGE: sodium dodecyl sulfate polyacrylamide gel electrophoresis
TMD: transmembrane domain
TPCK-trypsin: tosyl-phenylalanine chloromethyl-ketone treated trypsin
Wt: wild type
