# Supplementary material for: Hemagglutinin-esterase-fusion (HEF) protein of influenza C virus
Source: Protein Cell. 2015 Jul 28;7(1):28–45. doi: 10.1007/s13238-015-0193-x (PMC4707155; doi:10.1007/s13238-015-0193-x)
Supplement: Supplementary file 1 — Supplementary material 1 (PDF 620 kb) [file 13238_2015_193_MOESM1_ESM.pdf]

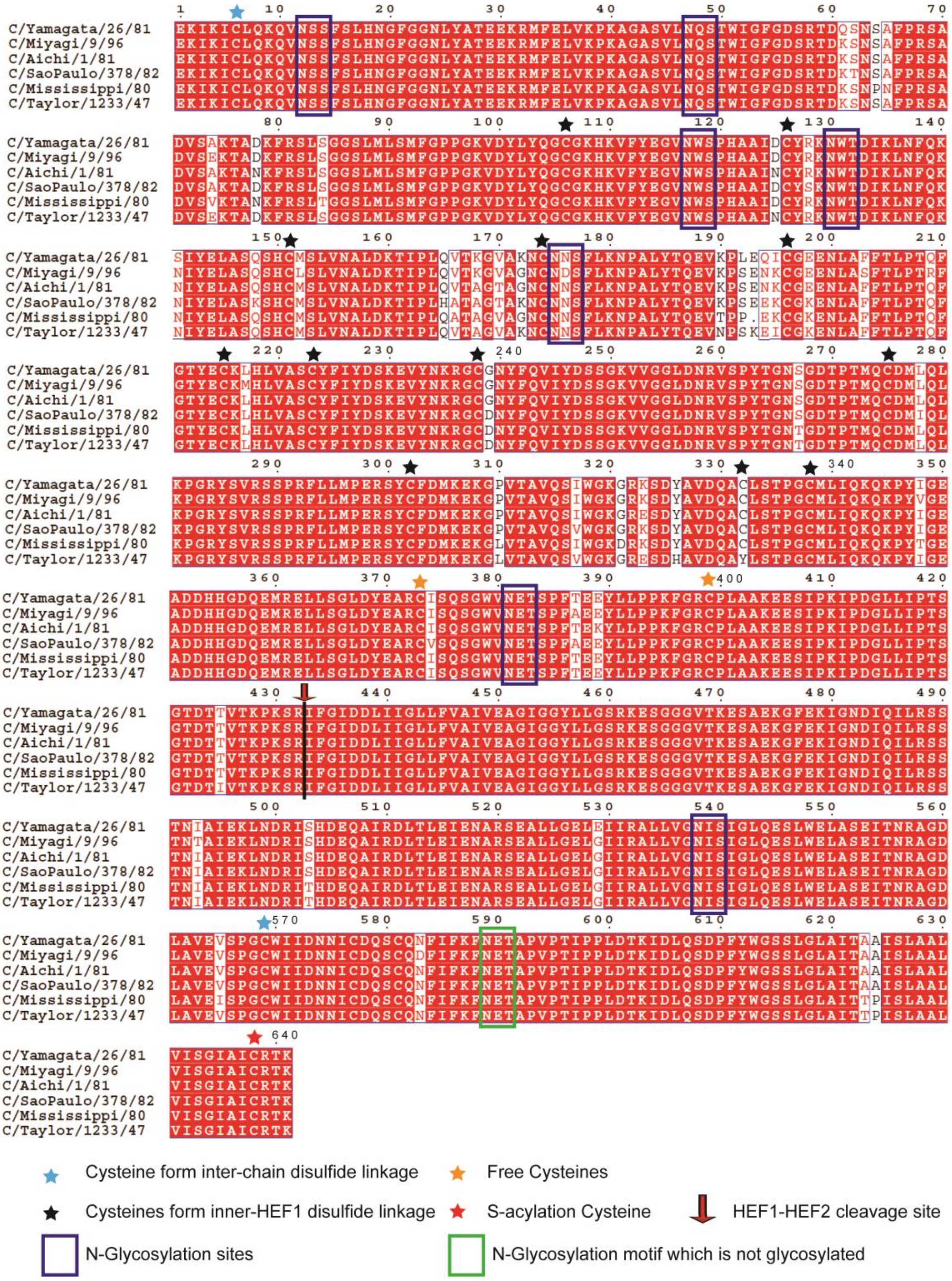

Supplementary table 1. Alignment of HEF proteins from six lineages of influenza C viruses. The cleavage site between HEF1 and HEF, cysteines and N-glycosylation motifs are highlighted as indicated.
